# Local Obtundents

**Published:** 1894-07

**Authors:** A. W. Haidle


### Local Obtundents.

BY A. W. HAIDLE, D.D.S.

Read before the 38th Annual Meeting of the Michigan Dental Association.

Local obtundents, or to use a more general term, local anæsthetics, are employed to temporarily abolish the sensibility of a part, and allow slight incisions or operations to be made painlessly.

Their effect is in some degree due to a paralysis of the end plates of the terminal branches of the cutaneous nerves, and possibly to some extent to a sedative action upon the blood vessels and tissues, analagous to that which is produced by rubbing or scratching, which gives temporary relief in itching. Local obtundents may be divided into two general groups:

1st. Agents which are applied or administered as drugs, either singly, or in combination with others.

2nd. Agents which are applied to produce thermal changes.

In the first division we find the following: aconite, atropine, campho-phenique, carbolic acid, chloral, chloroform, cocaine, menthol, morphine, opium, pyrethrum and veratrine.

As thermal agents we may employ: ice, ether spray, rhigoline, chloride of ethyl and chloride of methyl.

The greater number of the remedies mentioned in the first group are rarely used alone, being more frequently employed in combination with other drugs, and we find in our dental literature and text-books, and in the discussions of dental societies, numerous formulæ given, some of which are good, others inefficient, and, still, others which should be used with great caution. In order to construct a formula which shall prove efficient in obtunding the sensibility of the oral tissues, it will first be well to examine into the histology and physiology of these tissues, and into their relations with the general nervous and vascular systems. We find that the mucous membrane covering the alveolar process consists of an epithelial covering, lying upon a connective tissue stroma, the tunica propria. The epithelium is of the stratified squamous variety, and is continuous with the epidermis on the one hand and with the covering of the pharynx on the other. It occupies a place as regards power of absorption, midway between the two, having greater power of absorption than the epidermis, and less power than the mucous membrane of any part of the alimentary canal below the mouth. The tunica propria is composed of interlacing bundles of fibrous connective tissue, and possesses numerous simple papillæ, which encroach on the epithelial layer, but do not appear on the free surface of the mucous membrane.

The larger blood vessels lie within the sub-mucous tissue, and give off branches which extend through the deeper layers of the mucosa to the superficial portions of the connective tissue stratum ; on reaching the outer boundary of the latter the arteries break up into rich sub-epithelial capillary networks, and where papillæ are present, enter the minute elevations to supply their apices with terminal capillary loops. The nerve-fibers, largely of the medullated variety, accompany the blood-vessels, and end in a sub-epithelial plexus; and special terminations—the end bulbs—are found in the apices of some of the papillæ.

It will readily be seen that for the drug to accomplish its purpose of destroying sensibility, it must pass through the several layers of cells which make up the epithelium, and which range from the irregularly-shaped columnar and polyhedral cells, to the large, flat and scaly type so characteristic of the outer layers of stratified squamous epithelium, and must therefore possess in a marked degree either the power of penetration or the property of being easily absorbed.

By comparison with the mucous membrane lining the lower portions of the alimentary canal, it is found that while the mucous membrane of the stomach and of the intestines is especially adapted to the function of absorption as well as of secretion, that of the mouth is distinctively a secreting and not an absorbing tissue.

When, therefore, the power of penetration or the property of being easily absorbed are not inherent in the drug which is to form the *basis* of the formula, it becomes necessary to add a drug which will *aid absorption.*

Absorption can be aided in two ways: first, by increasing the circulation at the point of application, which can be accomplished by gently rubbing the part, or by the application of irritant substances; and second, by the use of an agent possessing endosmotic power. Such agents we have in chloroform and ether. Water, oils and resins do not have this property.

It is thought by some that alcohol possesses powers of endosmosis, but Waller has proved by experiments on the skin that this is not true. While it is as great a solvent of the sebaceous matter found in the skin as chloroform, it is not absorbed as quickly as this agent. Experiments on this point were made with iodide of potassium, which is a drug very readily detected in the urine. Waller, therefore, concludes that absorption is aided more by the endosmotic than by the solvent power of a drug.

If the basis of a formula is not sufficiently powerful in its action, an *adjuvant* should be added—a drug which will intensify or enhance the action of the basis. Such a drug we again find in the volatile liquids mentioned above, which are also aids to absorption. Heated applications act more energetically by increasing the circulation in the part.

It may be necessary to add a *corrective* to the formula. The basis may be too irritant or caustic and its harmful effects must be corrected. These effects may be either local or general, and the corrective used depends entirely upon the nature and action of the drug to be corrected. Only a few years ago chloral was extensively used as a local anæsthetic, and its irritant and caustic effects were demonstrated by the many cases of inflammation and sloughing of the part which resulted.

The local and the general effects of chloral are antagonized by carbolic acid, by atropine and by several others of the alkaloids. The nature of the action of these antagonistic drugs seems to be somewhat obscure. In commenting upon it, Brunton says: "Some regard the effect of one drug in counteracting another as a case of chemical combination or substitution, the second drug either becoming added on to a compound of the first with some of the tissues; or else displacing it from such a compound with the tissues. Others, again, think that no chemical action of this sort takes place, but that each drug acts upon the tissue by itself, —one, for example, exciting, and the other paralyzing."

Again, it may be necessary to add to the formula a *limiting agent*, a drug which will limit the endosmotic power of the basis. This may be accomplished in two ways: first, by the action of coagulants on the albumen of the protoplasm of the tissue cells; and second, by coagulative action on the blood capillaries and on the albumen of the serum contained in the tissues. Coagulation of the albumen of cell protoplasm may be produced by the use of such agents as carbolic acid, tannic acid, lead acetate, mercuric chloride, silver nitrate, copper sulphate, zinc chloride, etc., all of which combine with the albumen to form albuminates. Coagulation of the albumen of the serum in the tissues, and of the blood capillaries, may be produced by the use of astringents, including among those already mentioned as coagulants, others of the mineral and vegetable acids, alcohol, alum, and still other salts of the heavier metals.

In nearly all cases it will be necessary to employ a *diluent*, and the agent employed for this purpose should also be a *solvent*. The nature of the diluent must depend largely upon the nature of the basis, the diluents commonly used being chloroform, ether, alcohol, distilled water and glycerine.

Finally, it must be observed that the mixture is *antiseptic*, and that the agents employed are *compatible* with each other, to such a degree at least as not to prevent the accomplishment of the desired result. The mixture should be reasonably *stable*.

It will not be necessary to add to the formula a drug for each effect, for an agent may be employed to accomplish several results. As an example of such an agent chloroform may be mentioned; this may be employed as an adjuvant, an aid to absorption, a diluent, and it is also a solvent of almost unlimited power and scope. A good formula is given in Gorgas' *Dental Medicine*, which is as follows:

R. Tinctura aconiti....................j fl. ounce.
Menthol............................x grains.
Chloroformi...... ..................j fl. drachm.

M. S. Apply freely to gum about tooth for several minutes.

Aconite forms the basis and is a powerful nerve sedative. Its action when applied locally is to irritate the sensory nerve endings, causing a peculiar numbness and tingling, and afterward paralysis. Menthol is employed as an adjuvant, has marked anæsthetic action and is also antiseptic. The chloroform is the diluent and is an aid to absorption as well.

Another, and an excellent formula from the same source is as follows :

R. Chloroformi purificati.... ......

Tincturæ aconiti....................

Alcoholis, aa.......................j fl. ounce.

Morphinæ sulphat ..................vj grains.

M. S. Apply for one or two minutes to gum over root of tooth to be extracted.

A formula constructed by Dr. von Bonhorst contains :

R. Chloroformi.........................
Aetheris sulph.................. .......
Spiriti lavandulæ...................
Pyrethri (fl. ext.), aa............ j fl. ounce.

M. S. Apply for one or two minutes to gum over root of tooth to be extracted.

Among the drugs employed without combination with other drugs cocaine is the most important. An alkaloid occurring in colorless, transparent prisms, its salts are readily soluble in chloroform, ether, alcohol and water, and are rapidly absorbed.

The dose is gr. ss to gr. j. For epidermic application a solution in chloroform may be used varying in strength from a 6 per cent. to 20 per cent. solution. Dr. Hewitt, of Chicago, recommends the following formula for all operations about the gums:

R. Cocaine ..................................cxx grains.
Atropine ... ...............................$\frac{1}{10}$ grain.
Strophanthin...........................$\frac{1}{5}$ grain.
Naphthol hydrate ....................x grains.
Oil of cloves..................... ......
Oil of cassia...............................
Chloroform, aa.........................ij drachms.
Glycerine..................................j drachm.

The basis of this formula is cocaine, which used in this amount and proportion makes a 22 per cent. solution, very strong for epidermic application. It should be used with caution, and never be injected into the gums. This formula is deficient in correctives. Strophanthin is a very unstable and unreliable heart stimulant, and the quantity of atropine is too small. Carbolic acid would be more efficient as a corrective than naphthol.

Among the thermal agents mentioned, ice is never used as an anæsthetic, but is frequently employed to reduce inflammation, having a pronounced sedative effect. The ether spray is rarely employed, not being so effective as others of the thermal agents mentioned.

Rhigoline, one of the lighter products of petroleum distillation, is also rarely used because of its odor and the inconvenience of its application. It is a colorless liquid, of a disagreeable odor, quite volatile and inflammable, and produces an effect similar to the other volatile, freezing agents.

Chloride of ethyl is probably the most convenient and popular of the thermal agents of the present day. It is a clear, colorless liquid, possessing an agreeable, ethereal odor, and boils at 50° F. It is manufactured in France and is not found in the U. S. P. It is produced by the action of chlorine on ethyl alcohol, and is found in commerce in glass flasks which contain about three drachms. The method of application is to break the point of the flask, and after having protected the surrounding tissues, to direct the spray to the part to be anæsthetized. It evaporates very readily and causes intense cold, completely freezing the part, and rendering all slight operations painless. The best effect is produced when the flask is held at a distance of ten or twelve inches from the part, thus allowing the liquid to become vaporized before reaching the tissues. The patient should be instructed to breathe through the nose, and care should be taken that the application is not too prolonged, for if the part be too much deprived of blood, death, and subsequent sloughing of the tissue results. The anæsthetic stage is indicated by a whitening of the tissues and by the formation of ice crystals on the free margin of the gum.

Chloride of methyl, though not so extensively used, because of its greater comparative cost and the clumsiness of the apparatus in which it is contained, is a gas at 70° F. with a slight ethereal odor. It can be liquefied by pressure, and in commerce we find it in the liquid state held in copper flasks which contain about one quart.

As the chloride of ethyl, it is manufactured in France and is not found in the U. S. P. It is made from the remnants of beets used in the manufacture of beet sugar, extracting from these remnants the chlorate of tri-methylamine, which upon the addition of heat produces chloride of methyl. Its action differs from the action of the chloride of ethyl only in intensity, the former producing more cold and in less time than the latter.

DISCUSSION.--COCAINE.—LOCAL OBTUNDENTS.

Dr. Carrow, Ann Arbor, Mich.: My learned colleague has given us some very interesting facts concerning the action of cocaine upon the lower animals. But we must bear in mind the fact that there is a great difference between experimentation upon the lower animals in the laboratory and the practical use of the drug which the pharmacist furnishes us.

I would not disparage the value of such experiments, for they are of much service to us. But when we are told that a drug has a certain effect upon the lower animals we must not think that the same drug will necessarily produce a like effect on the human animal. I have found cocaine a safe and effective anæsthetic. I use a great deal of it in my practice. During the school year I have used, in my hospital practice, five ounces of cocaine. That is a large amount. In my private practice I, of course, do not use so much. Ordinarily I apply this in 4 per cent. solution, although I do use it in 6, 8, and in the nose, 10 per cent. solutions, being careful not to apply too much of the stronger solution. And yet, without exercising extreme care, either with respect to the patient or the strength of the solution, I can recall but one case where I have had trouble. In this case there was an idiosyncrasy against the use of this drug of which the patient was aware. She had a decided intermittent pulse. The operation was the removal of a truncated body which occluded the nostril. I gave her cocaine. I noticed none of the manifestations which my learned colleague has told us appear as a result of the administration of cocaine, except the irregular action of the heart. All other symptoms, dilation of the pupil, hurried respirations, etc., usually present under chloroform anæsthesia, I found in this case.

After an extensive use of this drug I am convinced that it is one of the best anæsthetic agents, at least for the ophthalmologist. I am partial to the large crystals of Parke, Davis & Co.

In operating upon the eye I drop the solution on the cornea. After four applications, made three minutes apart, two drops applied each time, the eye is sufficiently benumbed to enable me to open the eye and to perform the necessary operation without the slightest sensation on the part of the patient, and by this I mean absolutely does not feel it.

When operating upon small tumors, epithelioma of the tonsils or a small growth on the pharynx, I use about a quarter of a drachm of the 4 per cent. solution, going around every side of the tumor. The blood-letting which naturally follows the making of the incisions is sufficient to overcome what might otherwise be a dangerous tendency. I have been called in by other physicians in cases where there was exhilaration followed by extreme nervous condition and a low condition generally of the system, after the use of cocaine; and I have experienced the same thing in my own practice. But in all these cases a generous dose of whisky was suffficent to do away with all ill effects.

I think in dental practice the solutions used are not strong enough. Probably we will never arrive at the stage when anæsthesia, especially that produced by cocaine, will entirely benumb the tissues at the end of the fangs of the tooth, because we cannot reach the spot so as to deposit the cocaine there. But if the drug be applied about the superficial parts of the tooth I am sure the extraction can be accomplished without the patient having the slightest sensation. And if, instead of a one-half per cent. solution a drop or two of an 8 or 10 per cent. solution be applied to the surrounding surface before the rubber dam is adjusted, and as is so frequently the case, the thread or clamp jammed up into the alveolar process, operations would be painless and the irritation attendant upon such operations done away with.

As to the newer preparations of cocaine, which Dr. Cushny tells us are to come into general use, I would say the old preparation—hydrochlorate of cocaine—is good enough for my purpose; it is in itself a perfect drug.

Dr. Hoff, Ann Arbor, Mich.: Do you use a corrective remedy in connection with cocaine solution?

Dr. Carrow: I use simply a water solution except, in some cases, I accompany the hydrochlorate of cocaine with atropine; the effect of the atropine is greatly increased when the same quantity of hydrochlorate of cocaine is added to it.

C. P. Pruyn, D.D.S., Chicago, Ill.: Shortly after Nikolsky made his first experiments I procured some cocaine and began to use it locally, but with little effect as we used it at that time. I then began a series of experiments, using dogs for this purpose. The learned gentleman who has preceded me tells us that experiments upon dogs, and those upon man are not analogous. There are, however, many things that we need to know that can be learned only through experiments upon the lower animals. Many times I have seen symptoms that would have alarmed me had I not seen the same things when experimenting upon dogs.

Usually I procured two dozen dogs and paired them according to size and make-up. To one I gave cocaine; to its mate I administered the same dose of cocaine but first reinforced it with an antidote. For this purpose I found morphine best. I gave the morphine say twenty minutes before administering the cocaine.

I used small doses in all experiments as well as in practice. In one case five minims of a 4 per cent. solution produced marked dyspnœa. I now use altogether a 2 per cent. solution; I find I can accomplish with it all that I formerly accomplished with the 4 per cent. solution. I am surprised that Dr. Carrow should recommend such large doses. At Goshen, Indiana, death occurred from the administration of a little more than one grain.

Doubtless many of you have seen reports of this case. The gentleman was about fifty years old. He was a banker, a man of sedentary habits; he was not well preserved. He suffered from rheumatism and neuralgia and had chronic diarrhœa. He went to a dentist for an operation, as he was suffering so greatly from neuralgia. Seven or eight minims of a 2 per cent. solution of cocaine was given him and the operation performed satisfactorily. A month later he went to another dentist who gave him eight minims of a 4 per cent. solution and extracted several roots. In neither case were there toxicological effects. Ten days later, having other roots to be extracted, he went to the first dentist. This dentist had, in the mean time, seen a report of a formula published by Dr. Barrett, of Buffalo, which was as follows:

| | | |
|---|---|---|
| ℞. | Atropiae | $\frac{1}{10}$ grain |
| | Strophanthii | $\frac{1}{5}$ grain |
| | Cocaine mur | l grains |
| | Acidi carbolici | x grains |
| | Ol. caryophyli | 3 minims |
| | Aqua distil | j ounce |

He injected of that about ten minims, or possibly, a little more. Instantly there was a state of collapse and in twenty minutes the man was dead. Cases of this sort alarm us.

I took my little son, a lad of about ten years, to a rhinologist to have a simple operation performed on the nose. I took him about eleven o'clock in the morning, a time when digestion had taken place. The doctor applied a 10 per cent. solution of cocaine by means of a swab. The poisonous effects were manifested at once. The little lad was given aromatic spirits of ammonia and whisky. He soon recovered. A week later I again took him to the doctor, at the same hour of the day, but I gave him a cup of coffee and a sandwich as reinforcement. The same amount of the solution was used as before, but there were no ill effects. A short time after that he was taken again for a similar operation, at the same hour, but without the reinforcement. Everything was apparently all right. The operation was performed and the little fellow started from the office; then the poison began to take effect, and it was with the greatest difficulty that he reached my own office. The spasmodic effects were very marked in this instance. I applied the usual restoratives and resorted to artificial respiration.

In the name of my profession I would like to enter a protest against the claims made for cocaine by the gentleman who preceded me. It seems to me this drug is one that should be handled with a great deal of care. In dental practice the systemic effects of the drug are more marked than in cases of amputation of limbs, for instance, for the vital nerve centers are reached through the reflexes in operations about the mouth. All these patent nostrums, put forth as specifics for pain, have as their active principle cocaine. Somebody is going to suffer; thousands have already suffered. Note the way in which rhinologists use it. There is a case of chronic catarrhal condition; perhaps an operation has been performed. A prescription containing cocaine is written out. The patient takes it and experiences relief. He gets more of it, and then a little more, and thus is established a habit far more terrible than the opium habit.

Cocaine is a good remedy. I myself use it. But do not let us abuse the good things we have. Let all who intend to make use of this drug first experiment on the lower animals that they may become familiar with its action.

A French doctor is using cocaine in oil with a little acid for preservation. He thinks the cocaine is not so readily absorbed as when used with water. I have had no experience with this myself.

Dr. Cushny: I am gratified that so high a tribute should be paid to experimental pharmacology. The experimental pharmacologist was quite aware of the anæsthetic properties of cocaine twenty years before it came into use in medical practice. You will find it described in the text-book, for example, written in 1880, in which the author described fully the local anæsthesia produced by this drug; and although this book was written four years before the introduction of the drug, cocaine is there recommended in cases of operation upon the eye and upon the larynx.

I am much surprised that so small a dose as that given in the case at Goshen should have resulted in death. It is perhaps a question whether the drug used was pure or not, and whether death was not due to shock rather than to the drug. This same question arises in connection with the use of chloroform and ether, from the use of which so many deaths occur, especially in dentistry. Many times death is caused by shock. Complete insensibility is not always produced; the nervous system is weakened both by the drug and by the shock and death results.

I think different antidotes are indicated according to the stage of poisoning. If the exhilaration reaches a sufficiently high point a narcotic, such as chloral, is indicated ; in the stage of depression, undoubtedly the best drug is caffein injected subcutaneously or taken through the mouth, as suggested by Dr. Pruyn. This drug is a stimulant to the heart and to the nervous system.

The idea of using cocaine in oil is certainly a very curious one. It might retard the action of the cocaine, but before the drug takes effect it must be absorbed. I do not see that the oil is of special benefit.

Dr. Carrow: I feel that I must attempt to clear myself from the rather grave charge brought against me. Now, if I were to have anything done to my teeth I should want to go to such a man as Dr. Pruyn. He evidently is a close observer, and a careful and conscientious man—that is the kind of man I try to be in my own practice.

Now I do not want to advocate cocaine too strongly; I hardly know that I can. But I would like to ask this question: Because now and then death has occurred from the use of nitrous oxide, will you discard it? I think not. Because we have reports of deaths occurring from the use of chloroform, shall we empty our carboys of this drug and let our patients stand the shock occasioned the nervous system by the amputation of a limb or the removal of a tumor? I think not. Care should be exercised in using this drug, but I believe in giving it. In the case cited at Goshen, who knows what effect that particular combination of drugs may have had? I believe in a 4 per cent solution of cocaine. I make this solution fresh every morning. I take a drachm bottle which my little pipette is made to fit, and pour into it a drachm of saturated solution of boracic acid—which is as near water as you can get—then I turn out into my hand from my cocaine bottle what I think is about ιwo grains—I never measure it exactly—and put that into the bottle. If at night any of this solution is left it is thrown away and a fresh supply made the following morning. By exercising care in this way and reinforcing the patient, as suggested by Dr. Pruyn, I ams ure no ill effects will follow, and we may proceed with the full conviction that we are doing the best possible for our patients with the sanction which science, through experimentation upon the lower animals, has given us.

I am glad Dr. Pruyn spoke of the liability of contracting the cocaine habit—a most fascinating habit. I never write out a prescription for cocaine. I buy the drug myself and often the patient is not aware what he is taking; I am particular about this, especially if I think the person morally weak; for a strong man I would have no fear.

Dr. Taft: I would like to ask Dr. Pruyn the relative effect of cocaine when applied to the surface and when injected.

Dr. Pruyn: The effect of cocaine when applied locally is not so marked as when injected. In the latter case the effect is almost instantaneous.

Dr. Watling: Have you ever had trouble with sloughing at the point where the needle is inserted? I myself am a little shy of cocaine and have not used it much. But I have often seen this trouble when the drug has been injected; it is a question in my mind whether this is caused by the drug itself or by poison on the needle.

Dr. Pruyn: I have seen this effect but think it is due usually to dirt on the needle, or perhaps to septic poison on its point, not to the drug.

Dr. Watling: Is this more likely to occur on the gums than anywhere else?

Dr. Pruyn: No.

Dr. Taft: I do not know much about cocaine, but from the results produced when it was first introduced I have always regarded it as an unsafe agent to use miscellaneously. In the use of anything to which great danger is attached, the operator should be in the best possible condition, physically and mentally; the dentist because of business pressure or physical depression, is frequently not in a condition to make clear discriminations, and I think this is the cause many times of ill results. Where a dangerous agent is employed, as in the case of cocaine, special regard should be given to this point.

Dr. Birge: At no time have tools and appliances been so perfect as at present. Yet with all our improved machinery all our advanced, scientific methods of treatment, operations upon the teeth are attended with more or less pain. How to eliminate this is a subject which is receiving more attention from the profession than any other at the present day. And here the charlatans too are active; one cannot pick up a paper that has not one or more advertisements setting forth that by using some patent nostrum teeth may be extracted without pain.

The very able papers to which we have listened this morning and the discussions upon them will certainly enable us the better to cope with this matter. But all that has been said has been along the line of extraction. I think there are many other cases in which the dentist can make good use of cocaine. In the small cavities up under the gums it is well to touch the gum with a 10 or 20 per cent solution for a few moments before adjusting the rubber dam and the clamp; you will find there is not so much complaint about the clamp's hurting. When extracting the pulp I use a spoon-pointed syringe, injecting the cocaine in and around the pulp; then open with bur and extract with a barb. This can be done without pain to the patient and enables one to clean out the pulp and fill the tooth at one sitting. I have been afraid to inject cocaine directly for extraction of teeth. As Dr. Carrow has stated, it is hardly possible to inject cocaine deep down at the apex of the root because of the alveolar process; but injection into the gums will help the first taking hold of the tooth.

I have always held it a religious duty not to inject any preparation whose formula I did not know. It is not safe nor is it just to the patient.

I have used ethyl chloride a great deal and like it. Sometimes it leaves the gums intensely red and sore; a little vaseline rubbed on will relieve this. The following formula is one which, although I have not yet used sufficiently to speak with entire confidence, will, I think, prove very good:

| | | |
|---|---|---|
| R. | Sulph. atropine | $\frac{1}{2}$ grain |
| | Strophanthin | $\frac{1}{5}$ grain |
| | Carbolic acid (95 per cent.) | v gtts |
| | Hydrochlorate cocaine | xx grains |
| | Distil. water | q. s. j fl. ounce |

I inject four to six drops hypodermically.

I think the profession should "squelch" patent nostrums, and that all dental journals advertising such remedies should be taken off the list. Dentists can make up their own formulæ; if we cease buying these nostrums they will soon be withdrawn from the market.

DR. BETHEL, Kent, Ohio: I have always been very conservative in using drugs. Cocaine I consider a very dangerous article and think it should be used very cautiously. Indeed, it is a question in my mind whether it should be used at all hypodermically, especially above a 1 or 2 per cent. solution. I think, too, a third person should be present when it is administered, just as in the case of administering chloroform or nitrous oxide. Of course, there is a question as to whether the ill effects from the use of cocaine be due to the drug itself or carelessness in using it—whether to the impure article or septic poison due to an improperly kept syringe.

There is a great demand in these days for cheap things, and in order to cheapen some articles it is necessary to adulterate them; hence, many of our dental medicines are impure, cocaine among others. Of course, to obtain the best results our drugs must be pure. On the other hand, a drug may be pure when purchased but become impure through improper care. This is a point which is very important. If possible procure all drugs in original packages from reliable dealers.

All dental societies should take some action in regard to patent nostrums. To the gentleman who spoke a few moments ago in regard to striking from the list all journals advertising patent nostrums, I would say the editor is not always responsible for the action of the publishers in advertising matters, and in justice to the publishers, I might say that many times they would prefer not to print such advertisements—they often do not receive any pay for it.

There is a great demand for painless extraction. And undoubtedly, as Dr. Taft has said, most of the formulæ on the market for producing anæsthesia contain more or less cocaine. If a formula could be obtained that would be effectual and not contain this drug it would be a great boon to the profession as well as to suffering humanity.

I give a formula which, while I do not claim it to be the ideal anæsthetic, yet is one from which I have obtained good results:

| | | |
|---|---|---|
| R. | Carbolic acid crystals.................... | 10 grains |
| | Gum camphor.................................. | 8 grains |
| | Iodoform........................................ | $\frac{1}{2}$ drachm |
| | Listerine ........................................ | 2 ounces |
| | Pond's extract of hamamelis............ | 2 ounces |

In the preparation of this the carbolic acid and gum camphor should be mixed first. (Gum camphor when mixed with carbolic acid crystals melts down and forms a clear liquid.) To this add the iodoform followed by the listerine and hamamelis. The bottle should be shaken occasionally for, at least, twenty-four hours. The liquid is then filtered. Iodoform has considerable anæsthetic properties; camphor is said to increase solubility of the iodoform. Not all the iodoform is dissolved and what is taken up is held mechanically in the mixture, but enough is incorporated by constant shaking.

Dr. Hoff: I have thought that if we had some formula upon which we all could report, our attention would in this way be called to the question of formula-making and in this way we might be able to do something toward doing away with quack nostrums. I offer the following for test:

| | | |
|---|---|---|
| R. | Cocaine........................................ | $\frac{1}{2}$ grain |
| | Morphine sulph ................................ | $\frac{1}{4}$ grain |
| | Atropine (tablet)................................ | $\frac{1}{80}$ grain |
| | Carbolized water ($\frac{1}{2}$ per cent.)........... | xxx gtts |

As you will observe, this formula contains both morphine and atropine. Morphine acts more promptly when introduced as a preliminary drug. All three ingredients are local anæsthetics. According to Dr. Cushny's statements the whole of this preparation could be safely used. I scarcely ever use more than five or six drops for six teeth.

Dr. Taft: Every well-informed, self-respecting dentist who feels prepared for his profession should have a thorough knowledge of the things entering into his equipment. He who makes use of patent nostrums has not that knowledge. In a general way he may know of what the compound is composed, but the exact formulæ is not disclosed, for obvious reasons, and he who uses them acknowledges his own weakness; he is worshiping unknown gods.

In relying too much upon anæsthetics we are weakening our influence over our patients. There is a certain psychical power which every dentist and physician, too, has or ought to have over his patients, which causes that person to have implicit faith in whatever you may do or say. When such patients come to you and the question is asked: "Do you wish to take an anæsthetic?" reply, "Oh, no. I do not suffer very much under your hands." If we resort too freely to anæsthetics this confidence on part of the patient is destroyed. Therefore, I would say to you, especially the younger members of the profession, bear in mind that you have a power which you ought to cultivate and use for the benefit of your patient, and not throw aside for these things out of the use of which so much iniquity, danger and harm are likely to arise.

Dr. Jackson: There is one thing which has not been spoken of. How many of you are careful to use pure distilled water? It is just as easy to introduce poison into the system through the water used as in any other way. It is well, too, even if you have disinfected the instrument, to dip its point, just before using, into carbolic acid.

Dr. Pruyn was asked to give his method of injecting.

Dr. Pruyn: I do not know that my method is very different from that employed here at the University of Michigan. I use a hypodermic glass barrel syringe with a minim gauge on the flange. This gauge I consider very important. On my own instrument there is a little bar on the piston which can be set at any point on the gauge. By using this sort of instrument there is no danger of injecting more of the fluid than is intended. I have cut off the point about one half; the ordinary hypodermic syringe point is too long for our work. A fine point is best, but not one so fine as to be too elastic.

I think the point of Dr. Jackson's a very good one. Even when sterilized water is used it is well to dip the point of the instrument into some antiseptic solution, for disease germs floating about in the air may have been deposited there. Bichloride solution, or a week solution of cinnamon water are, I think, preferable to carbolic acid. The cinnamon solution need not be strong enough to act as an escharotic solution is apt to weaken it; for that reason cinnamon is perhaps better for this purpose.

Dr. Parker: I have found very good results from the injection of *aqua pura.* I have tried this remedy in perhaps half a dozen cases of broken roots. It works admirably. No bad effects follow its use, and according to the patients, operations under it are painless. Probably this is the result of the psychological effect spoken of by Dr. Taft.

Dr. Pruyn: I have used this same valuable prescription in many cases and I could not distinguish the slightest difference between its effect—so far as sense of pain is concerned—and those of a 4 per cent solution of cocaine. I have had no sloughing nor ill effect of any kind. I have imagined that better results were obtained when warm water was used.

Dr. Parker: I have thought it best to use water just as cold as I could.

Dr. Pruyn: I have thought that perhaps the effect from use of water came from pressure upon the nerve terminal filaments. There may be something in this.

A Member: A young lady presented herself to me to have some roots extracted. She had been told to ask for cocaine or something of that kind. I offered nitrous oxide but she did not wish to take that. I applied extract of hamamelis and extracted three teeth *painlessly.*

